# Exploring the use of adjusted body mass index thresholds based on equivalent insulin resistance for defining overweight and obesity in UK South Asian children

**DOI:** 10.1038/s41366-018-0279-7

**Published:** 2018-12-13

**Authors:** Mohammed T. Hudda, Angela S. Donin, Christopher G. Owen, Alicja R. Rudnicka, Naveed Sattar, Derek G. Cook, Peter H. Whincup, Claire M. Nightingale

**Affiliations:** 1 Population Health Research Institute, St George’s, University of London, London, UK; 2 Institute of Cardiovascular and Medical Sciences, University of Glasgow, Scotland, UK

**Keywords:** Epidemiology, Cardiovascular diseases

## Abstract

**Background:**

Body mass index (BMI) overweight/obesity thresholds in South Asian (SA) adults, at equivalent type-2 diabetes risk are lower than for white Europeans (WE). We aimed to define adjusted overweight/obesity thresholds for UK–SA children based on equivalent insulin resistance (HOMA-IR) to WE children.

**Methods:**

In 1138 WE and 1292 SA children aged 9.0–10.9 years, multi-level regression models quantified associations between BMI and HOMA-IR by ethnic group. HOMA-IR levels for WE children were calculated at established overweight/obesity thresholds (at 9.5 years and 10.5 years), based on UK90 BMI cut-offs. Quantified associations in SA children were then used to estimate adjusted SA weight-status thresholds at the calculated HOMA-IR levels.

**Results:**

At 9.5 years, current WE BMI overweight and obesity thresholds were 19.2 kg/m^2^, 21.3 kg/m^2^ (boys) and 20.0 kg/m^2^, 22.5 kg/m^2^ (girls). At equivalent HOMA-IR, SA overweight and obesity thresholds were lower by 2.9 kg/m^2^ (95% CI: 2.5–3.3 kg/m^2^) and 3.2 kg/m^2^ (95% CI: 2.7–3.6 kg/m^2^) in boys and 3.0 kg/m^2^ (95% CI: 2.6–3.4 kg/m^2^) and 3.3 kg/m^2^ (95% CI: 2.8–3.8 kg/m^2^) in girls, respectively. At these lower thresholds, overweight/obesity prevalences in SA children were approximately doubled (boys: 61%, girls: 56%). Patterns at 10.5 years were similar.

**Conclusions:**

SA adjusted overweight/obesity thresholds based on equivalent IR were markedly lower than BMI thresholds for WE children, and defined more than half of SA children as overweight/obese.

## Introduction

Body mass index (BMI), the most widely used method for assessing adiposity, underestimates total body fat (BF) in South Asian (SA) people [1]. Several UK studies have shown that at equivalent type-2 diabetes (T2D) risk, BMI levels are markedly lower in SA adults compared to white European (WE) adults [2–4], suggesting that BMI thresholds for defining overweight and obesity in SA adults should be lower than those for WEs [1–5]. However, no equivalent thresholds have been developed in children. Although BMI thresholds based on equivalent T2D risks would be difficult to quantify in children, an alternative would be to use equivalent levels of insulin resistance, a strong and consistent precursor of T2D risk both in adults [6, 7] and in children [8], in whom consistent ethnic differences are observed [9]. Here we explore the use of this approach to estimate adjusted BMI thresholds for defining overweight and obesity in UK–SA children.

## Research design and methods

In the Child Heart and Health Study England (CHASE), a school-based study of 9.0–10.9 year UK children of different ethnic origins [9] conducted between 2004 and 2007, height was measured with a portable stadiometer (Chasmors Ltd, London, UK) and weight with an electronic digital scale (Tanita Inc, Tokyo, Japan). Female pubertal status was assessed using Tanner breast development staging [10]. Participant ethnicity was defined using the ethnicity of both parents or if not available, parentally-defined child ethnicity. Serum insulin and plasma glucose were measured in fasting blood samples [9], allowing Homeostasis Model Assessment insulin resistance [11] (HOMA-IR) to be determined.

Statistical analyses were performed using Stata v14. HOMA-IR was positively skewed and log transformed (natural log transformation) for analysis. Multi-level regression models were fitted using the residual maximum likelihood approach to assess the associations between log HOMA-IR (dependent variable) and BMI in both ethnic groups for both boys and girls. Models were adjusted for age (continuous), sex, ethnic group and month of measurement (fixed effects) with a random effect to allow for clustering of children at school level. Two-way interaction terms between BMI and both sex and ethnicity were tested using the Wald test at the 5% significant level. A statistically significant interaction was detected between BMI and ethnic group (P_interaction_ = 0.01), which was included in the model as a fixed effect. Standardised residual plots were used to assess the model fit.

To determine adjusted BMI thresholds for SA children, we obtained sex-specific thresholds for overweight and obesity in WEs from UK90 growth charts [12]. The coefficients from the multi-level regression models (described above) were used to estimate levels of HOMA-IR in WEs at the UK90 overweight and obesity threshold levels. The same regression model coefficients were then rearranged to estimate BMI levels in SAs corresponding to the same HOMA-IR values. This process was embedded within a bootstrapping procedure to obtain 95% bootstrap confidence intervals (95% BCI) for these estimates (described in the Appendix).

## Results

In all, 2430 children (49.7% boys) aged 9–10 years without diabetes provided complete data, including 1138 WE and 1292 SA children. Characteristics of the analysis sample are summarised in Supplementary Table 1. WE children were on average taller and heavier than their SA counterparts but had lower HOMA-IR levels (Supplementary Table 1).

### Associations between BMI and log HOMA-IR

After adjusting for age, sex and month of measurement, each 1 kg/m^2^ increase in BMI was associated with a 9.2% (95% CI: 8.2–10.1%) increase in HOMA-IR in WE and a 10.8% (95% CI: 10.0–11.7%) increase in SA children. Histograms and normal plots of the standardised residuals from the regression models did not show any departures from normality, and there was no evidence of residual curvature when standardised residuals were plotted against fitted values.

### BMI thresholds for overweight and obesity in SA children at equivalent HOMA-IR

#### Nine-year-olds (central age 9.5 years)

**Fig. 1:**
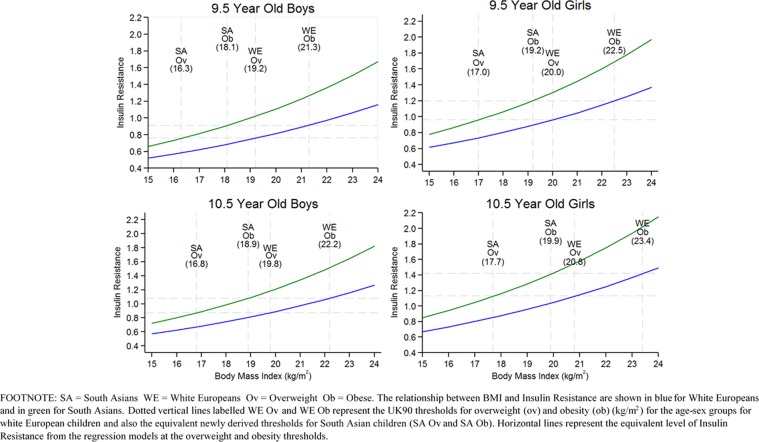
Associations between BMI and HOMA insulin resistance, showing adjusted BMI thresholds for overweight and obesity in South Asian Boys (left) and girls (right) aged 9.5 (top) and 10.5 (bottom) years based on equivalent HOMA-Insulin Resistance levels in White Europeans

Current overweight and obesity thresholds for WE boys are 19.2 kg/m^2^ and 21.3 kg/m^2^, respectively. The equivalent HOMA-IR levels corresponding to these thresholds were 0.8 and 0.9, respectively. At these HOMA-IR levels, overweight and obesity thresholds for SA boys were lower by 2.9 kg/m^2^ (95% BCI: 2.5–3.3 kg/m^2^) and 3.2 kg/m^2^ (95% BCI: 2.7–3.6 kg/m^2^), respectively (Fig. 1 & Supplementary Table 2). Overweight and obesity thresholds for WE girls are 20.0 kg/m^2^ and 22.5 kg/m^2^, respectively. Equivalent levels of HOMA-IR were 1.0 and 1.2; corresponding overweight and obesity thresholds for SA girls were lower by 3.0 kg/m^2^ (95% BCI: 2.6–3.4 kg/m^2^) and 3.3 kg/m^2^ (95% BCI: 2.8–3.8 kg/m^2^), respectively (Fig. 1 & Supplementary Table 2).

#### Ten-year-olds (central age 10.5 years)

Current overweight and obesity thresholds for WE boys are 19.8 kg/m^2^ and 22.2 kg/m^2^, respectively and for girls are 20.8 kg/m^2^ and 23.4 kg/m^2^, respectively. At equivalent HOMA-IR levels, overweight and obesity thresholds for SA boys were lower by 3.0 kg/m^2^ (95% BCI: 2.5–3.4 kg/m^2^) and 3.3 kg/m^2^ (95% BCI: 2.8–3.8 kg/m^2^), respectively and for SA girls were lower by 3.1 kg/m^2^ (95% BCI: 2.7–3.6 kg/m^2^) and 3.5 kg/m^2^ (95% BCI: 2.9–4.0 kg/m^2^), respectively (Fig. 1 & Supplementary Table 2).

Among SA children, the prevalences of overweight and obesity using the adjusted BMI thresholds increased markedly, so that more than half of both 9 and 10 year old SAs were classified as overweight or obese, compared with between a quarter and a third using conventional BMI thresholds (Supplementary Table 3). The exclusion of girls with evidence of pubertal development did not materially affect these results.

## Conclusions

This study quantified ethnic differences in the associations between childhood BMI and HOMA-IR (a precursor of T2D risk) to examine the feasibility of developing ethnic-specific BMI thresholds for overweight and obesity for UK–SA children based on equivalent HOMA-IR to those in WE children. In SAs, HOMA-IR levels were markedly higher and the associations between HOMA-IR and BMI were stronger, so that BMI thresholds for overweight and obesity at equivalent HOMA-IR were markedly lower.

To our knowledge, this is the first report to estimate overweight and obesity thresholds in UK–SA children based on equivalent HOMA-IR. On this basis, BMI thresholds defining overweight and obesity in UK–SA children aged 9–10 years  would be ~3 kg/m^2^ lower. These differences in BMI thresholds between SA and WE children are appreciably larger than those in our earlier UK study based on equivalent total BF (~1.1 kg/m^2^ for 4–12 year olds) [13]. However, the present results are consistent with reports from the Netherlands and New Zealand, which suggested that, based on equivalent BF levels, BMI thresholds for SA children should be 2–3 kg/m^2^ [14] or 3–4 kg/m^2^ lower [15].

Thus derivation of ethnic-specific BMI thresholds for SA children based on equivalent HOMA-IR (reflecting their higher emerging T2D risk at any given BMI level) would be feasible, though further validation over a wider age-range would be needed. However, the observed prevalences of overweight and obesity in SA children using these adjusted thresholds are extremely high, with more than half of SAs aged 9–10 years identified as overweight or obese. These findings emphasize the major population health challenge represented by overweight, obesity and T2D in the UK–SA population. Although individually based identification and management of overweight and obese children could play a role in prevention, focussed population-wide strategies for controlling weight gain in SA children, emphasizing the maintenance of healthy diets with appropriate energy intakes coupled with sustained physical activity through childhood and beyond, will be particularly important.

## Supplementary information

Supplementary Material
