# Supplementary material for: Exploring the use of adjusted body mass index thresholds based on equivalent insulin resistance for defining overweight and obesity in UK South Asian children
Source: Int J Obes (Lond). 2018 Dec 13;43(7):1440–3. doi: 10.1038/s41366-018-0279-7 (PMC6451638; doi:10.1038/s41366-018-0279-7)
Supplement: Supplementary file 1 — Supplementary Material [file 41366_2018_279_MOESM1_ESM.docx]

SUPPLEMENTARY TABLE 1: CHARACTERISTICS OF STUDY SAMPLE BY ETHNIC GROUP AND SEX

|  | **White European** | | | **South Asian** | | |
| --- | --- | --- | --- | --- | --- | --- |
|  | **Boys N = 635** | **Girls N = 635** | **Overall N = 1270** | **Boys N = 729** | **Girls N = 713** | **Overall N = 1442** |
| Age (years) | 10.0 (9.7-10.2) | 10.0 (9.7-10.3) | 10.0 (9.7-10.3) | 9.9 (9.7-10.2) | 10.0 (9.7-10.3) | 10.0 (9.7-10.2) |
| Height (cm) | 139.4 (135.0-143.3) | 139.1 (134.3-143.7) | 139.3 (134.7-143.4) | 138.2 (134.1-142.6) | 138.7 (134.0-143.9) | 138.4 (134.0-143.2) |
| Weight (kg) | 34.4 (29.9-39.9) | 34.5 (29.8-40.5) | 34.5 (29.9-40.2) | 32.8 (28.2-39.8) | 34.3 (29.1-40.9) | 33.5 (28.7-40.5) |
| BMI (kg/m^2^) | 17.6 (16.1-20.0) | 17.8 (16.2-20.4) | 17.7 (16.1-20.2) | 17.2 (15.4-20.0) | 17.6 (15.8-20.5) | 17.4 (15.5-20.3) |
| Fasting insulin (mU/L) | 5.7 (3.9-8.1) | 6.7 (4.7-9.2) | 6.2 (4.3-8.6) | 7.2 (4.9-10.8) | 8.9 (6.1-13.0) | 7.9 (5.5-11.9) |
| HOMA Insulin Resistance | 0.7 (0.5-1.0) | 0.9 (0.6-1.2) | 0.8 (0.5-1.1) | 0.9 (0.6-1.4) | 1.1 (0.8-1.6) | 1.0 (0.7-1.5) |

Values shown are Median (Lower quartile – Upper quartile)

SUPPLEMENTARY TABLE 2: BMI THRESHOLDS (KG/M^2^) FOR OVERWEIGHT AND OBESITY (AND 95% BOOTSTRAP CI) IN SOUTH ASIAN CHILDREN BASED ON EQUIVALENT HOMA-INSULIN RESISTANCE LEVELS TO THOSE IN WHITE EUROPEAN CHILDREN, BY AGE AND SEX

|  |  | 9.5 Years Old | | | 10.5 Years Old | | |
| --- | --- | --- | --- | --- | --- | --- | --- |
|  |  | White European | South Asian | Difference between White European and South Asian | White European | South Asian | Difference between White European and South Asian |
| Boys | Overweight | 19.2 | 16.3 (15.9 – 16.7) | 2.9 (2.5 – 3.3) | 19.8 | 16.8 (16.4 –17.3) | 3.0 (2.5 – 3.4) |
|  | Obese | 21.3 | 18.1 (17.7 – 18.6) | 3.2 (2.7 – 3.6) | 22.2 | 18.9 (18.4 – 19.4) | 3.3 (2.8 – 3.8) |
|  |  |  |  |  |  |  |  |
| Girls | Overweight | 20.0 | 17.0 (16.6 – 17.4) | 3.0 (2.6 – 3.4) | 20.8 | 17.7 (17.2– 18.1) | 3.1 (2.7 – 3.6) |
|  | Obese | 22.5 | 19.2 (18.7 – 19.7) | 3.3 (2.8 – 3.8) | 23.4 | 19.9 (19.4 – 20.5) | 3.5 (2.9 – 4.0) |

For White Europeans the BMI thresholds come from the age and sex specific UK90 growth charts

SUPPLEMENTARY TABLE 3: PREVALENCE (%) OF OVERWEIGHT AND OBESITY IN WHITE EUROPEAN AND SOUTH ASIAN CHILDREN BASED ON UNADJUSTED BMI THRESHOLDS AND ADJUSTED BMI THRESHOLDS FOR SOUTH ASIANS, BY AGE AND SEX

|  |  | PREVALENCE % | | | | | |
| --- | --- | --- | --- | --- | --- | --- | --- |
|  |  |  | 9.5 Years Old  N=1,276 | |  | 10.5 Years Old  N=1,154 | |
|  |  | White  European  N=299 | South Asian  *Unadjusted Threshold*  N=348 | South Asian  *Adjusted Threshold*  N=348 | White  European  N= 271 | South Asian  *Unadjusted Threshold*  N=290 | South Asian  *Adjusted Threshold*  N=290 |
| Boys  N=1,208 | Overweight | 15 | 13 | 19 | 16 | 13 | 21 |
|  | Obese | 14 | 18 | 41 | 16 | 13 | 33 |
|  | Overweight-Obese | 29 | 31 | 61 | 31 | 27 | 54 |
|  |  |  |  |  |  |  |  |
|  |  | White  European  N=290 | South Asian  *Unadjusted Threshold*  N=339 | South Asian  *Adjusted Threshold*  N=339 | White  European  N= 278 | South Asian  *Unadjusted Threshold*  N=315 | South Asian  *Adjusted Threshold*  N=315 |
| Girls  N=1,222 | Overweight | 18 | 16 | 24 | 12 | 15 | 20 |
|  | Obese | 11 | 11 | 33 | 10 | 9 | 30 |
|  | Overweight-Obese | 29 | 27 | 56 | 22 | 24 | 51 |

Sex-specific BMI thresholds for overweight and obesity obtained from the UK90 BMI charts at 9.5y and 10.5y

APPENDIX: DESCRIPTION OF BOOTSTRAP TECHNIQUE USED TO OBTAIN ESTIMATES OF ADJUSTED BMI THRESHOLDS AND THE ASSOCIATED IMPRECISION FOR SOUTH ASIAN CHILDREN

To determine the adjusted BMI thresholds for SA children:

1. Sex-specific thresholds for overweight and obesity in WEs at ages 9.5 and 10.5y were obtained from the UK90 growth charts (12)
2. 1000 randomly generated bootstrap samples, were generated separately for each sex. Each sample was the same size as our original sample and generated by sampling with replacement, and stratified by school.
3. For each of the 1000 bootstrap samples a multi-level regression model was fitted in which ln (HOMA-IR) was regressed on BMI, age and ethnic group, separately for each sex. An interaction term was included between BMI and ethnic group while school was fitted as a random effect.
4. The HOMA-IRs corresponding to the sex and age specific thresholds of BMI from [1] were estimated for WEs using the estimated “fixed effect” coefficients from regression equations in each bootstrapped sample in [3].
5. The fixed effect regression coefficients from each bootstrapped iteration in [3] were then rearranged to estimate the corresponding BMI cut points for SA children which corresponded to the HOMA-IR levels in WE children estimated in [4].
6. The 1000 BMI cut points in SA children generated in [5] were then averaged to obtain a bootstrapped mean estimate the adjusted cut points in SAs and the associated 95% bootstrap reference range provided the bootstrapped confidence interval (BCI), separately for each sex.
